# Breeding Sex Ratios in Adult Leatherback Turtles (*Dermochelys coriacea*) May Compensate for Female-Biased Hatchling Sex Ratios

**DOI:** 10.1371/journal.pone.0088138

**Published:** 2014-02-04

**Authors:** Kelly R. Stewart, Peter H. Dutton

**Affiliations:** 1 Marine Mammal and Turtle Division, Southwest Fisheries Science Center, National Marine Fisheries Service, National Oceanic and Atmospheric Administration, La Jolla, California, United States of America; 2 The Ocean Foundation, Washington D. C., United States of America; University of Texas Arlington, United States of America

## Abstract

For vertebrates with temperature-dependent sex determination, primary (or hatchling) sex ratios are often skewed, an issue of particular relevance to concerns over effects of climate change on populations. However, the ratio of breeding males to females, or the operational sex ratio (OSR), is important to understand because it has consequences for population demographics and determines the capacity of a species to persist. The OSR also affects mating behaviors and mate choice, depending on the more abundant sex. For sea turtles, hatchling and juvenile sex ratios are generally female-biased, and with warming nesting beach temperatures, there is concern that populations may become feminized. Our purpose was to evaluate the breeding sex ratio for leatherback turtles at a nesting beach in St. Croix, USVI. In 2010, we sampled nesting females and later sampled their hatchlings as they emerged from nests. Total genomic DNA was extracted and all individuals were genotyped using 6 polymorphic microsatellite markers. We genotyped 662 hatchlings from 58 females, matching 55 females conclusively to their nests. Of the 55, 42 females mated with one male each, 9 mated with 2 males each and 4 mated with at least 3 males each, for a multiple paternity rate of 23.6%. Using GERUD1.0, we reconstructed parental genotypes, identifying 47 different males and 46 females for an estimated breeding sex ratio of 1.02 males for every female. Thus we demonstrate that there are as many actively breeding males as females in this population. Concerns about female-biased adult sex ratios may be premature, and mate choice or competition may play more of a role in sea turtle reproduction than previously thought. We recommend monitoring breeding sex ratios in the future to allow the integration of this demographic parameter in population models.

## Introduction

Animals with temperature-dependent sex determination (TSD) may exhibit biases in the primary sex ratio that may be skewed toward female or male depending on the species and the location. For example, in Nile crocodiles (*Crocodylus niloticus*), where females are produced at low incubation temperatures, embryos examined from natural nests in Zimbabwe were highly skewed to female [1]. A review of crocodilian sex ratios reported that alligators and crocodiles generally exhibit female-biased ratios [2]. However, over several years and at several sites, juvenile alligator sex ratios were slightly male-biased [3]. These studies underscore the importance of studying primary sex ratios both regionally and temporally. For species that depend on environmental temperatures for sex determination of offspring, there is concern that climate change may put some species at risk [4], [5], and that primary sex ratios may become even more skewed [6], [7] unless species are able to adapt either spatially or temporally [8].

However, perhaps more important than the primary sex ratios for the persistence of a species or the resilience of that species to climate change are the ratios of males to females that are available to breed, also known as the operational sex ratio (OSR). The OSR directly affects the mating system, because sexual selection theory predicts that the more numerous of the sexes cannot afford to be choosy with mates [9]. Additionally, OSR affects courtship as a form of competition [10], and other mating characteristics like sexual dimorphism [11] and extra-pair copulation [12] have all been linked to the OSR. In wandering albatross (*Diomedea exulans*), there is a primary sex ratio bias toward males that persists through adulthood, despite juvenile males having lower survivorship rates and with the species having varying levels of maternal and paternal mortality depending on the sex of the chicks [13]. However, because some of the adult males are post-reproductive, the OSR becomes more balanced [13]. Ideally, as in the Weimerskirch et al. (2005) study, understanding how the primary sex ratio relates to the OSR and how it varies over an animal's lifetime allows us to better understand population dynamics and assess vulnerability to climate change.

One of the taxa reported to be at risk of warming temperatures are sea turtles, all of which exhibit TSD. The primary sex ratios of hatchlings are generally highly female-biased [14], although some exceptions have been noted [15]. While extensive work has been done to determine primary sex ratios in sea turtles, partly because sea turtles are accessible on nesting beaches, little is known about the OSRs. Sea turtles have a promiscuous mating system - both polyandry and polygyny have been documented [16] however little information is available about mate choice or competition. Making estimates of OSR is made more challenging by the difficulty in encountering male turtles at the breeding grounds; they are rarely ever seen, while females come ashore to lay eggs and are identified individually.

The purpose of our study was to determine the breeding sex ratio for the leatherback (*Dermochelys coriacea*) population at St. Croix, USVI. We identified and counted breeding males by using maternal and hatchling genotypes to infer the genotypes of individual males that had successfully bred in a particular year.

## Materials and Methods

### Ethics statement

All procedures involving hatchling leatherback sea turtles described herein were rigorously reviewed and subsequently approved by the US Fish and Wildlife Service (Sandy Point National Wildlife Refuge Special Use Permit #41526-2010-002) and the Department of Planning and Natural Resources of the US Virgin Islands (Department of Fish and Wildlife Permit #STX-020-10) prior to sample collection. All hatchlings were collected within the boundaries of Sandy Point National Wildlife Refuge (St. Croix, US Virgin Islands). As hatchlings emerged naturally from nests, they were collected and sampled. Individual turtles were sampled by being placed on a sanitized polyethylene board (4″×4″×0.13″). The biopsy site on the trailing edge of the front flipper was disinfected with isopropyl alcohol (70%) and a single skin sample was taken using a sterilized 2-mm biopsy punch (Integra™ Miltex® Inc., York, PA USA). Samples were stored in a saturated salt solution. Following sampling, each biopsy site was treated with a hemostatic agent (styptic pencil – aluminum sulfate 56%) to prevent bleeding. After hatchlings were sampled, they were maintained for observation in a shallow rectangular plastic bin containing a layer of damp sand until nightfall. Once we ensured that hatchlings were behaving naturally (orienting toward a bright horizon and crawling energetically), and within two hours of being sampled, they were released just above the high tide line and observed as they crawled to and entered the water.

### Field site

Detailed information on the field site at Sandy Point National Wildlife Refuge (SPNWR) and methods of genetic sample collection may be found in Stewart and Dutton (2011)[17]. Briefly, each year from March through July (since 1981), nesting female leatherbacks are monitored, tagged and their nests triangulated. In addition to being identified through flipper tags and Passive Integrated Transponder (PIT) tags, each female is sampled and fingerprinted genetically. The presence of a unique mtDNA haplotype demonstrates that turtles nesting here exhibit natal homing [18], [19]. With this in mind, beginning in 2009 we have been conducting a genetic mass-tagging project with hatchling leatherbacks at SPNWR to derive an estimate of age to maturity. Because each nest is marked and linked to an individual female, we are able to collect live hatchlings as they emerge after identifying their mothers. Genetic samples are then taken [20] from every hatchling.

Building on the proof of concept developed in Stewart and Dutton (2011)[17] where we were able to evaluate multiple paternity and reconstruct paternal genotypes (and thus identities) from 12 known mothers and their hatchlings, we expanded our assessment to include as many females for which we had at least 10 hatchlings sampled. In addition to nests that had fewer than 10 hatchlings, some female's nests were not available for sampling (hatchlings emerged either prior to or after our main sampling season) and some female's nests failed to hatch or were washed away. From June 22 to August 8, 2010, we collected and sampled 3747 hatchlings from 151 nests belonging to 91 females (multiple nests per female). Although 91 females nested in 2010, we were able to examine paternal contributions to the hatchlings from 58 of those females, for the reasons listed above.

### DNA analysis

Using an X-tractor Gene robot and standard manufacturer protocols (Qiagen Inc., Valencia, CA, USA), we extracted total genomic DNA from each hatchling sample. We then amplified the DNA in a 25-µl volume reaction using Polymerase Chain Reaction (PCR) [21] and ABI 2720 or Bio-Rad PTC 100 thermal cyclers. We used seven microsatellite markers with the following published reaction schemes: D1 [22], LB133, LB141, LB142 and LB145 [23] and CcP5H07 and CcP5C08 [24]. The PCR product for each sample was analyzed using ROX500 fluorescent size standard (PE Applied Biosystems, Foster City, CA, USA) in an ABI Prism 3730 Genetic Analyzer. We used Genemapper 4.0 (Applied Biosystems, Foster City, CA, USA), with manual verification, to score alleles. To evaluate samples for contamination, we ran both positive and negative controls with every DNA extraction plate; negative controls were also run for every PCR reaction and analyzed at the same time as the hatchling samples. In addition, we ran replicates of samples (re-amplified and rescored) to determine the genotyping error rate and then corrected any that were wrongly called. Micro-Checker v 2.3.3 [25] was used to assess allelic stutter, large allele dropout, and null alleles in the hatchling genotype dataset.

### Statistical analysis

We first identified the maternal allelic contribution to each hatchling genotype and then identified the paternal alleles for each hatchling set using GERUD1.0 [26]. Once all possible genotypes of the fathers were identified, we conducted the exhaustive search option for all the genotypic combinations to identify the minimum number of fathers that would explain the hatchling genotype dataset. From the alleles that were assigned to the father, we reconstructed the complete genotype, thus identifying individual males.

We assessed Hardy-Weinberg equilibrium and heterozygosity at 7 polymorphic loci for the nesting females and generated allele frequencies using GENEPOP version 4.1.10 [27]. We calculated the probability of detecting multiple paternities with individual loci (d) and for all seven loci combined (D) [28] as well as determining the probability (Q) that any two females or any two males shared an identical genotype for all loci [29].

## Results

Of the 58 females (of 91 nesters in 2010) that we evaluated in this study, we determined genotypes for 662 of their hatchlings. The number of hatchling samples per female that were successfully amplified and genotyped ranged from 6 to 21 (average  = 11.4). The overall genotyping error rate for both hatchlings and mothers combined was 3.4% (38 allele calls incorrect out of a total of 1112). With Micro-Checker [25], we found that there was no evidence of scoring error due to stutter, no null alleles and no large allele dropout. When multiple paternity was detected with the initial 6–10 hatchlings per female, it was sometimes necessary to genotype more hatchlings to be able to determine the exact paternal genotypes. Once genotyping was complete for all females and their hatchlings, we determined that in three cases, nests had been incorrectly assigned to females in the field; this was determined because genotypes of the hatchlings and the females did not match. We therefore excluded these three cases from the paternal genotype reconstruction.

For the nesting turtles, summary statistics are provided in Table 1. The number of alleles for the seven microsatellite loci ranged from 5 to 14, and overall (all 7 loci) they were in Hardy-Weinberg equilibrium (p = 0.6); the range for expected heterozygosity was 0.64 to 0.90. The probability that any two females shared an identical genotype across seven loci was 1.01×10^−8^ (Q) and the probability of detecting multiple paternity across all loci was 0.998 (D).

**Table 1:** For 51 nesting females, the number of alleles per locus, the expected (He) and observed heterozygosities (Ho), Hardy-Weinberg p-value (HW), the probability of two females having the same genotype at a locus (q) and the probability of being able to detect multiple paternity at each locus (d) are given.

Locus	# alleles	He	Ho	HW	q	d
**D1**	13	0.90	0.94	0.56	2.2×10^−2^	0.78
**LB133**	10	0.67	0.73	0.96	1.4×10^−1^	0.46
**LB141**	6	0.69	0.78	0.65	1.5×10^−1^	0.44
**LB142**	5	0.64	0.73	0.39	1.6×10^−1^	0.43
**LB145**	6	0.69	0.67	0.63	1.4×10^−1^	0.46
**CcP5H07**	14	0.89	0.98	0.99	2.5×10^−2^	0.76
**CcP5C08**	12	0.86	0.80	0.04	4.0×10^−2^	0.70

Of the 55 females that were matched definitively to their nests, 42 had mated with only one male each (single paternity), nine had mated with at least two males each and four had mated with at least three males (rate of multiple paternity  = 23.6%). Using GERUD1.0 and the hatchling and maternal genotypes, we were able to resolve the paternal genotypes for the mates of 46 females. In some of the multiple paternity cases, it was possible to detect at least 2 or 3 fathers, but genotypes could not be resolved so the number of fathers would be considered a minimum. During this process, we identified 47 individual males, resulting in a breeding sex ratio of 1.02 males: 1 female. Of the 47 males, Males #1, 2, 10, 32, 40 and 41 had mated with two females each, while one male (Male #17) had mated with three separate females. The remaining 40 males had mated with just one female each. The probability of 2 males sharing an identical genotype was 7.92×10^−9^.

## Discussion

Although primary sex ratios for leatherbacks are often highly female-biased [30], [31], [32], and the only in-water study of foraging adult leatherbacks reported catching nearly twice as many females as males (1.86 females: 1 male) [33], we found that the breeding sex ratio was balanced for the nesting females that we sampled at St. Croix in 2010. We evaluated just over 50% of the females that nested in that season and found that the number of breeding males (47) was slightly higher than the number of breeding females (46). While the true OSR (the ratio of available breeding males to available breeding females) cannot logistically be calculated because not all turtles available for breeding can be counted in the water, we suggest that the breeding sex ratio derived from successful matings may begin to serve as a proxy for this important population parameter. While the breeding sex ratio from our study may be considered preliminary, we recommend that future studies examine all nesting females to determine every mating pair to provide a more robust estimate of breeding sex ratio for the population within a given year.

When we compared the males identified in this study with those from a previous multiple paternity study at the same site [17], in which 17 males were responsible for hatchlings from 12 females in 2009, we found that two males had been reproductively successful in consecutive years. We have now identified 62 individual males (for 58 females) in this rookery over two years and conclude that males are not lacking in number, at least in this population and potentially in others. However, a longer term study of adult male and female identification would be helpful in discovering the true adult sex ratio. In a study of green turtles nesting in Cyprus [34], the OSR was reported to be 1.3 males for every female over a three-year period, and in hawksbills in the Seychelles (35), 47 males were found to have mated with 43 females (1.1 males; 1 female). Studies into breeding sex ratios, OSR and breeding periodicity contribute greatly to our understanding of sex ratio variation in adult sea turtles.

Both the OSR and the breeding sex ratio may vary between years for a given population because those parameters depend on how many individuals of each sex make the migration to waters adjacent to the nesting beach. Breeding periodicity is much easier to determine for female sea turtles through intensive tagging programs and direct observation, while males are more difficult to encounter. Due to physiological constraints, females skip nesting for one or more years following a nesting season and only return to the breeding grounds once they have stored enough energy for egg production as well as for the migration from distant foraging areas. Since males have significantly lower energetic costs, they could be expected to breed more frequently. Evidence from satellite telemetry suggests that this is indeed the case; male leatherbacks are able to make annual migrations between breeding and foraging areas [36] and thus are likely to have a shorter breeding periodicity than females. In Greece, based on satellite tracking and tagging data, Hays et al. (2010)[37] concluded that male loggerhead turtles (*Caretta caretta*) were migrating to the nesting beach 2.6 times as frequently as females [37]. In contrast, 97% of male green turtles (*Chelonia mydas*) in Cyprus mated only once within a three-year period [34]. While a few males in that study did return more than once over three years, none of the females returned in subsequent years. It is likely that breeding periodicity varies by sex and species at different nesting beaches.

Critical questions remain regarding the relationship between the primary sex ratio and the OSR in sea turtles. If primary sex ratios are highly skewed to female, is there differential mortality for male vs. female hatchlings? Are there differences in breeding periodicity or differences in maturity or longevity for males and females? To date there is none of this information known for leatherbacks as very few juveniles are ever seen and the age to maturity and longevity is not known. In a male-biased adult population of Nazca boobies (*Sula granti*) [38], the adult sex ratio could not be explained by the sex ratio of fledglings, which was balanced. Instead, sex-specific differential mortality in the post-fledging period was likely responsible [38]. In the terrapin (*Malaclemys terrapin*), differing ages at maturity for males and females resulted in a male-biased OSR in adults [39].

Understanding how climate change may affect both hatchling and adult sex ratios for sea turtles will be important for assessing their capacity for persistence. With growing evidence that the breeding sex ratio or OSR may compensate or mitigate female-biased hatchling and juvenile sex ratios, some focus should be placed on understanding the mating system in more detail, particularly in relation to mate choice, female choice and competition. Some studies have suggested that the breeding adult density affects levels of competition and multiple paternity [35], [40]; with more breeding adults, there is a higher level of multiple paternity. It will be increasingly important to understand the contribution of males for any population, particularly for species with TSD.

Sea turtles may mitigate extreme female-biased primary sex ratios that might arise through climate change by adjusting nesting behavior either in space (moving to cooler areas) or by nesting earlier or later in the season [41]. If turtles do alter their behavior in response to warming temperatures, over time we should expect to see hatchling sex ratios that are similar to what we see now (female-biased overall) and therefore worries about adult populations becoming entirely feminized may be unfounded.

To date, the majority of studies on sex ratios in sea turtles have focused on hatchling sex ratios because of the ease of access to hatchlings, a long-standing interest in TSD, and the motivation to understand as much as possible about the incubation environment. However, we recommend focusing more attention on calculating breeding sex ratios and estimating OSR for these threatened and endangered species, as the adult sex ratio has the potential to directly impact the viability of populations. This study provides an excellent model for evaluating adult breeding sex ratios in sea turtle populations, requiring only that the mother is known and that ∼10–20 hatchlings and the mother are genotyped at several informative markers. With a growing number of markers available for assessing sea turtle genetics, studies such as this one should be straightforward, provided that facilities and funds are available. Identifying breeding males from genetic fingerprints of mothers and their hatchlings fills a critical information gap in our knowledge of sea turtle population dynamics and allows us to set better recovery goals by incorporating information for both sexes in recovery plan objectives. In addition, accounting for male turtles in the population allows for full evaluation of breeding assemblages, thus improving life-table parameter estimates for modeling purposes. The conservation value of being able to individually identify males for a census, determine breeding periodicity and evaluate male reproductive success cannot be understated.
